# Familial Essential Thrombocythemia With Novel MPL L502G and G208K Mutations

**DOI:** 10.7759/cureus.23220

**Published:** 2022-03-16

**Authors:** Matthew Rendo, Christian Cavacece, Chung-ting J Kou, Bradley W Beeler, Joshua Fenderson

**Affiliations:** 1 Hematology and Oncology, Brooke Army Medical Center, San Antonio, USA; 2 Internal Medicine, Brooke Army Medical Center, San Antonio, USA

**Keywords:** inherited mpn, thromboembolism, jak2 negative essential thrombocythemia, mpl, familial essential thrombocythemia, essential thrombocythemia

## Abstract

Familial essential thrombocythemia is characterized by the inheritance of germline mutations to progeny, thereby increasing the risk for the development of essential thrombocythemia. Here, we present two cases of young women who developed thromboembolic phenomena, one of whom with an ischemic event despite adequate anticoagulation. Through extended mutational testing, both were characterized as having novel mutations in the myeloproliferative leukemia virus (MPL) gene, and both individuals have fathers being treated for essential thrombocythemia. This case provides insight that in familial essential thrombocythemia, there remain uncharacterized mutations in this inherited conditional landscape.

## Introduction

Essential thrombocythemia (ET) is a subset of the broader category of chronic myeloproliferative neoplasms (MPNs) that typically result in elevated blood counts. MPNs were once thought to be caused solely by de novo mutations, but recent genetic analysis has revealed a familial component. Up to 10% of previous cases once thought to be sporadic show a heritable family history [1]. This number could be an underestimation as variable penetrance has been shown and normal blood counts could preclude an indication for additional genetic testing. The complications from MPNs, specifically ET, can lead to devastating thrombotic events if not properly treated. We present two cases of patients found to have novel mutations, varying platelet levels, and clinical scenarios consistent with a form of hereditary thrombocythemia. Both initially presented with thromboembolic phenomena with no obvious provoking factors.

## Case presentation

Case 1

**Figure 1:**
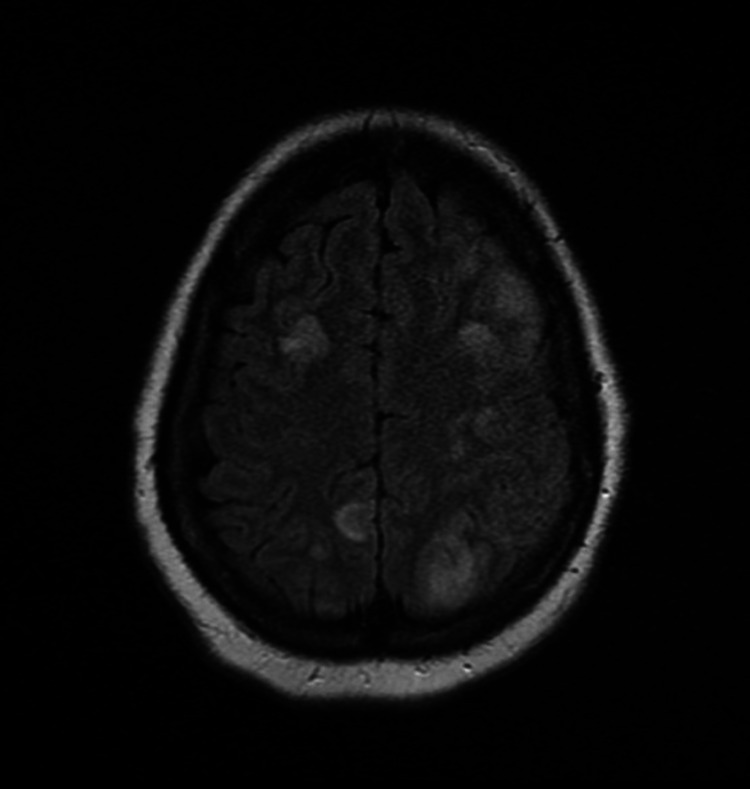
T2-weighted MRI axial imaging revealing multiple bilateral infarcts in the cerebral cortex

A 44-year-old female presented with an unprovoked saddle pulmonary embolism (PE) causing pulseless electrical activity with multiple bilateral cerebral infarctions (Figure 1). The PE was treated with a tissue plasminogen activator, and the patient recovered with no residual deficits beyond a mild anoxic brain injury. Full cardiology evaluation to include a trans-esophageal echocardiogram, telemetry, and extended cardiac rhythm monitoring yielded no cardiologic etiologies. During the hospital stay, the patient was also found to have iron deficiency anemia, reported a history of menorrhagia, and was treated with an iron infusion. She was referred to hematology for anticoagulation management without definitive hypercoagulable etiologies elucidated and continued anticoagulation with rivaroxaban. She underwent a hysterectomy for fibroids.

Two years later, she presented again to the hematology clinic with hemoglobin (Hgb) of 15.3 g/dL, hematocrit of 47.2%, ferritin of 469 ng/mL, and platelet count of 344 per uL. JAK2 mutational analysis and hereditary hemochromatosis (HH) mutation assessing for an underlying MPN or HH that was previously masked by menorrhagia returned negative. A sleep study exhibited obstructive sleep apnea, and the patient initiated nocturnal continuous positive airway pressure (CPAP).

Reflex testing for thrombopoietin (TPO) receptor mutations, encoded by the myeloproliferative leukemia virus (MPL) oncogene gene, exhibited a positive mutation. The patient mentioned that her father was also being treated for essential thrombocythemia. Subsequent chromosome sequence analysis (CSA) revealed a heterozygous variant of unknown significance with the genomic change Chr1(GRCh37):g.43814969_43814970delinsGG, a variant predicted to result in a single amino acid substitution of leucine for glycine at codon 502 in exon 10 of the MPL gene. According to the Human Gene Mutation Database (HGMD), this variant has not been reported as being associated with a clinical condition.

Subsequent labs showed an improvement in hemoglobin to 14.5 g/dL after CPAP compliance and platelets remained in the normal range. A bone marrow biopsy revealed slight hypercellularity at 80% with maturing trilineage hematopoiesis and mildly increased megakaryocytes. There was no evidence of dysplasia nor the typical morphologic “staghorn megakaryocytes” seen in ET. During regular follow-ups, the patient had developed diffuse pruritus, palmar erythromelalgia, and a transient episode of monocular vision loss. Brain MRI revealed no acute infarcts, and the patient exhibited symptomatic improvement with monthly phlebotomy and the addition of aspirin.

Case 2

**Figure 2:**
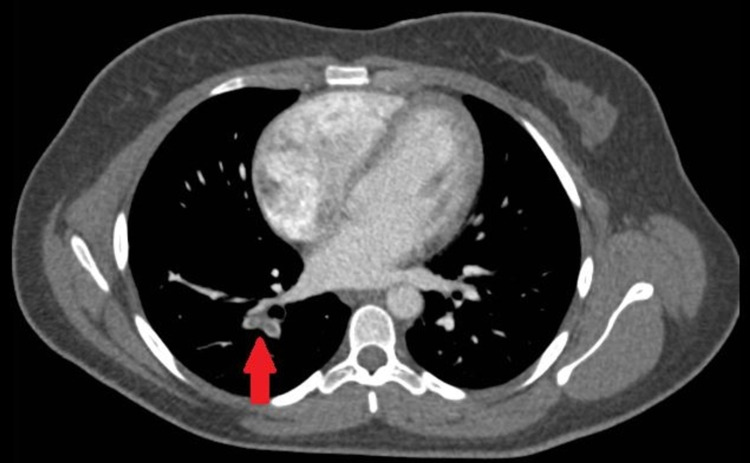
Axial CT imaging revealing pulmonary embolism in the right lower lobe segmental pulmonary arteries (arrow)

The second patient is a 19-year-old female who presented to an ER with left-sided chest pain and cough. CT imaging revealed multiple PEs in the left inferior segmental pulmonary arteries. She had no provoking factors at that time nor other complications and was discharged on rivaroxaban with Hgb 14.9 g/dL and platelet count of 402 per uL. She presented six weeks later with right lateral chest wall pain and shortness of breath. Her Hgb was 6.6 g/dL and platelets were 485 per uL. The patient informed that after starting rivaroxaban, she developed menorrhagia. CT imaging showed a new pulmonary embolism in the right lower lobe segmental pulmonary arteries, with decreased clot burden in the left inferior segmental pulmonary artery branches (Figure 2).

The patient received a packed red blood cell transfusion and was initiated on a heparin drip. She exhibited no signs of heart failure nor hypoxia and initiated norethindrone for menses suppression. Further labs were notable for ferritin of 6.8 ng/mL, and she received an iron transfusion. She was bridged to warfarin and followed up one month later with Hgb 10.8 g/dL, ferritin 36.1 ng/mL, iron saturation 25%, and platelet count 566 per uL. The hypercoagulable workup was unrevealing to include: protein C, protein S, antithrombin III, factor V Leiden, prothrombin gene mutations, lupus anticoagulant, cardiolipin antibodies, and beta-2 glycoprotein antibodies. Persistent thrombocytosis and a report that the patient’s father was being treated for ET prompted testing for the JAK2, MPL, CALR, and JAK2 exon 12-15 mutations, which were also all negative.

Subsequent CSA revealed a heterozygous variant of unknown significance with the genomic change Chr1(GRCh37):g.43805172C>A, a variant predicted to result in a single amino acid substitution of glutamine to lysine at codon 208 in exon 4 of the MPL gene. This variant had also not been reported as associated with a clinical condition per the HGMD. A subsequent bone marrow biopsy revealed normocellular marrow with trilineage hematopoiesis and mild megakaryocytic hyperplasia without atypia nor dysplasia. The patient continues on warfarin without any secondary complications or recurrent thromboembolic events.

## Discussion

Several proteins have been implicated in the pathogenesis of ET, all within the megakaryocytopoiesis pathway. In addition, there are several growth factors involved, the major one being thrombopoietin (TPO). Overactivation of this pathway, leading to ET, can be either primarily from clonal progenitor cells or from secondary inflammation leading to persistent stimulation. Secondary thrombocytosis is much more common and is believed to account for up to 96.7% of thrombocytosis cases, the majority triggered by infection [2]. However, a difference has been observed in regards to venous thromboembolic events, with a significantly higher proportion seen in the primary thrombocytosis group [2]. Roughly half of all ET diagnoses have an underlying JAK2 V617F mutation as the driving force behind platelet overproduction. The rest of the mutations are scattered throughout CALR, MPL, and other genes that also act on the JAK-STAT pathway of platelet development. The mechanism of MPL mutations leading to ET involves the receptor for TPO being mutated into a constitutively active form. This occurs independently of TPO and leads to the overproduction of platelets [3].

The first patient presented does not follow the typical WHO diagnosis of ET with a platelet count not greater than 450 per uL, whereas the second patient had more of a classic presentation (albeit initial mutation analysis was negative). Both patients’ MPL mutations, L502G and G208K, are not explicitly described in the literature. Similarly, both patients have an affected first-degree relative, an identified germline MPL mutation, experienced menorrhagia, and significant unprovoked thrombotic evens. More research is coming to fruition regarding the familial aspect of ET and all MPNs. A 12-fold elevated risk was seen in a Swedish epidemiological study among first-degree relatives with ET and about 8% of Philadelphia chromosome-negative MPNs have documented familial clustering [4-5]. Although the relative’s exact mutation was unknown in both cases, recent studies have shown the familial clustering of MPNs does not always exhibit classic Mendelian inheritance and is thought to arise from shared risk factors and weak penetrance [6]. This provides evidence to suggest screening in first-degree relatives with mildly elevated, or even normal, blood counts may have a role. However, it is yet to be determined as the most effective way to screen, as CSA is cumbersome, expensive, and not readily available. There are continued ongoing discoveries of mutations other than the typical V617F on JAK2, of which subsequent positive findings may be difficult to interpret the clinical significance of in an asymptomatic patient. The ideal situation would be the detection of high-risk patients with the early initiation of antiplatelet or anticoagulant medications and preventing future thrombotic events.

In regards to treatment for patient one, much of the data was extrapolated from previous studies on the more prevalent and documented S505N mutation. Both this and our patient’s mutation are within the transmembrane domain of the TPO receptor. Several studies have confirmed that patients with the S505N mutation are at increased risk for thrombotic complications, especially those who were not taking antiplatelet agents [7]. This patient also had several symptoms of platelet activation (e.g. aquagenic pruritus, monocular vision loss), and the patient was started on aspirin in addition to the rivaroxaban she was already taking. Furthermore, the familial studies did not show the anti-thrombotic benefit of anti-proliferation medications. This differs from previous evidence that favors the use of hydroxyurea in high-risk patients, with a history of thrombotic events and sporadic ET [8]. Patient one never had an elevated platelet count and had symptomatic control with the initiation of phlebotomy and aspirin, negating the need for anti-proliferative medications. A bone marrow biopsy was done in both patients to exclude a clonal proliferation or malignancy. In the limited case reports of hereditary thrombocytosis, no cases of evolution into acute leukemia were observed [9]. Notwithstanding, the more common V617F mutation has been directly linked to the progression of hematologic neoplasms [10].

As for patient two, a similar approach was attempted by using existing data on closely located mutations [11]. However, there is limited information regarding similarly located mutations of the TPO receptor as seen in this patient’s mutation. Notwithstanding, it is hypothesized that this mutation also contributes to a constitutively activated TPO receptor and platelet activation, leading to a propensity for thromboembolic phenomena. Since the patient had a recurrent PE on rivaroxaban, warfarin was initiated. This patient has not exhibited further symptoms of platelet activation since the initiation of warfarin, thus a joint decision was made for pursuing warfarin monotherapy.

## Conclusions

Familial ET is an entity that has increasing evidence detailing the elevated risk of inheritance in first-degree relatives. We have presented two cases of novel mutations that have not been described in the literature and could potentially highlight the underreported nature of familial ET. A high index of suspicion for familial MPNs should be suspected in patients with an affected first-degree relative and an unprovoked thrombotic event. This series emphasizes the importance of thorough family history in regards to hematologic abnormalities.
